# The Efficacy of Manipulation with Distension Arthrography to Treat Adhesive Capsulitis: A Multicenter, Randomized, Single-Blind, Controlled Trial

**DOI:** 10.1155/2022/1562358

**Published:** 2022-02-13

**Authors:** Yayun Zhang, Ruirui Xue, Zhengyi Tong, Mengchen Yin, Yiqun Yu, Jie Ye, Jinhai Xu, Wen Mo

**Affiliations:** ^1^Department of Orthopaedics, Longhua Hospital, Shanghai University of Traditional Chinese Medicine, Shanghai, China; ^2^Department of Orthopaedics, Huangpu Branch, Shanghai Ninth People's Hospital, Affiliated to Shanghai Jiaotong University School of Medicine, Shanghai, China

## Abstract

**Objective:**

To determine whether arthrographic distention combined with manipulation for frozen shoulder provides additional benefits.

**Methods:**

A total of 180 participants from five clinical centers with pain and stiffness in predominantly 1 shoulder for >3 months entered the study, and 165 completed the study. The control group was treated with arthrographic distention alone, and the treatment group underwent manipulation after resting for 5 minutes following arthrographic distention. Patients were followed up at the one and two weeks and at three and six months. For the clinical evaluation, shoulder-specific disability measure (SPADI) score, the visual analog scales (VASs) for pain, and range of active motion were used.

**Results:**

83 patients out of 90 in the treatment group and 82 out of 90 in the control finished the entire study period. SPADI, VAS, Constant-Murley (CM), and range of motion (ROM) were improved after treatments in both groups. The statistical differences were not observed in the CM, adduction, internal rotation, and posterior extension function between groups (*P* > .05) after the first treatment. And the statistical differences were not observed in the internal rotation, the extorsion, and posterior extension function (*P* > .05) after the second treatment.

**Conclusion:**

Distention arthrography plus manual therapy provided faster pain relief, a higher level of patient satisfaction, and an earlier improvement in AROM of the shoulder than distention arthrography alone in patients with frozen shoulder.

## 1. Introduction

Frozen shoulder (FS) was first defined by Codman in 1934, and it is characterized by shoulder pain and active dysfunction caused by inflammation of the soft tissue around the shoulder, also known as adhesive capsulitis [1]. FS is a common cause of shoulder pain, affecting 2% to 5% of the general population [2]. The prevalent age of FS is more than 40 years, with a higher incidence in women than men [3]. The etiology of FS is still controversial and includes inflammatory responses, local microcirculatory disorders, fibroplasia, neurogenic inflammation, degenerative changes, and paralysis of the shoulder muscles [4, 5].

Research on the use of CAM in different musculoskeletal disorders has aroused widespread interest [6–8]. The treatment objectives for FS are to relieve pain, regain shoulder motion, and restore function. Recommended treatments for FS include physical therapy, analgesia, and gentle exercise [9]. Various therapies have different effects at different stages of the disease. For example, oral nonsteroidal anti-inflammatory drugs increase the risk of adverse events such as gastrointestinal bleeding, ulcers, and perforation. Patients with FS causing severe pain or limited range of motion (ROM) are treated with intra-articular injections, distension arthrography, manipulation under anesthesia, and surgery. However, intra-articular corticosteroid injections only achieve short-term pain relief [10]. Distension arthrography was first proposed in 1982 and is currently recommended as the preferred treatment for FS due to its effectiveness in relieving pain and improving active dysfunction [11]. Manual therapy improves pain, ROM, muscular strength, and level of functional activity [12].

In recent years, we have explored a treatment method for FS. All patients treated for FS in our institution receive the Three-step and Nine-therapy manipulative therapy (involving kneading, drawing, and rubbing the shoulder) combined with distension arthrography. Due to the absence of clinical evidence, we designed a multicenter, randomized, single-blind, controlled trial to evaluate the safety and clinical efficacy of this new therapeutic method for FS. The purpose of this research is to determine whether arthrographic distension combined with manipulation of a frozen shoulder provides additional benefits.

## 2. Materials and Methods

### 2.1. Study Design

**Figure 1:**
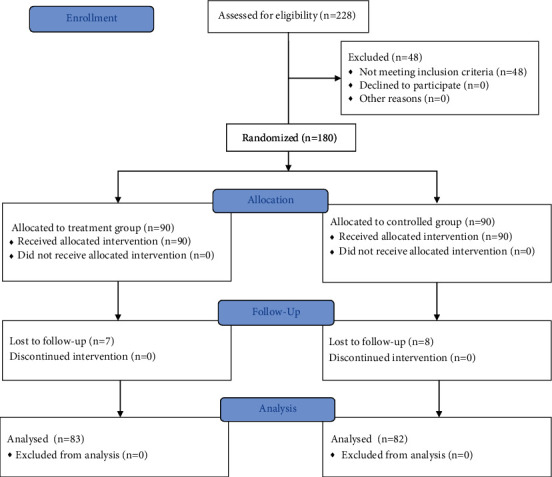
CONSORT project overview.

This was a prospective, multicenter, randomized, controlled trial performed to evaluate the efficacy of distension arthrography plus manipulative therapy in the management of FS. The study was conducted in accordance with the principles of the Declaration of Helsinki [13]. Patients were treated from April 2013 to March 2015. All participants had a diagnosis of FS confirmed by orthopaedic surgeon. All patients underwent diagnostic shoulder x radiograph and magnetic resonance imaging. The study was approved by the ethical committee of the Longhua Hospital (No. 2013LCSY061). Written informed consent was obtained from all subjects prior to study participation. The clinical trial was registered in the Chinese Clinical Trial Registry (ChiCTR-INR-17012945). The study was conducted in accordance with the CONSORT checklist (Figure 1).

The following six hospitals participated in the study: Longhua Hospital, Shanghai University of Traditional Chinese Medicine; hospital of traditional Chinese medicine, Shanghai Qingpu district; Dachang community health service center, Shanghai Baoshan district; Waitan community health service center, Shanghai Pudong New Area district; Shanggang community health service center, Shanghai Pudong New Area district; and Nanmatou community health service center, Shanghai Pudong New Area district.

### 2.2. Sample Size

The sample size was calculated based upon the ability to detect a 10-point difference in the Shoulder Pain and Disability Index (SPADI), which is reported to indicate a clinically important improvement (or worsening) of shoulder function [14]. Considering a 0.05 two-sided significance level in each group, a power of 90% to detect a difference in mean SPADI values of ≥10 with a standard deviation of ≤15, and an allocation ratio of 1 : 1, 39 participants were required in each group. Allowing for a dropout rate of 20%, a total of 180 participants were included (90 participants in each group) [15]. Each participating clinical center treated 30 patients, comprising 15 in the control group and 15 in the treatment group.

### 2.3. Randomization and Allocation

Randomization lists were computer-generated using the SPSS 20.0 software to randomize participants to the two groups using a web-based randomization system managed by an independent third-party clinical research organization (Institute of Basic Research in Clinical Medicine, China Academy of Chinese Medical Science). At each of the six participating hospitals, the randomized allocations were kept in opaque envelopes and were opened individually for each patient who agreed to participate in the study. No other stratification or blocking procedure was used. The sponsors had no role in the design and conduct of the study, the collection, management, analysis, or interpretation of the data, or the preparation, review, or approval of the manuscript. The study was conducted in accordance with the trial protocol. Patients and investigators were not blinded to the type of treatment.

### 2.4. Eligibility Criteria

The inclusion criteria were as follows: (1) age 40 years or older, (2) symptoms lasting more than 1 month, (3) no abnormal findings on radiographic study and the type of FS is primary, and (4) limited ROM of the shoulder in at least two directions (less than 120° of forward flexion and less than 50% of the internal and external rotation on the normal side). All the patients include in this study have a confirmed diagnostic of adhesive capsulitis [16, 17].

The exclusion criteria were as follows: (1) history of major shoulder injury or surgery, (2) systemic inflammatory joint disease, (3) polymyalgia rheumatica, (4) treatment with corticosteroids in the previous 3 months, (5) poorly controlled diabetes, (6) pregnancy, (7) allergy to local anesthetic, (8) contraindications to corticosteroid injections, (9) osteoarthritis of the glenohumeral joint, (10) rotator cuff disease, (11) fibromyalgia, and (12) unwillingness or inability to participate in follow-up procedures.

### 2.5. Interventions

#### 2.5.1. Distension Arthrography

All injections were performed by a senior physiatrist who had received clinical rheumatology training and had many years of experience performing intra-articular shoulder injections.

The coracoid was palpated, and its inferior margin edge was selected as the needle entry site. The treatment group was treated with arthrographic distension, followed by 5 minutes of rest, and then manipulation therapy. The control group underwent arthrographic distension without manipulation. All patients received a total of two arthrographic distension treatments with a 1-week interval. The skin around the marked injection site was cleaned with antiseptics. A needle attached to a 50 ml syringe was then inserted into the glenohumeral joint. After the clinician detected the popping of the needle through the anterior capsule in conjunction with the loss of normal injection resistance, 35 ml of distension fluid (5 ml of 2% lidocaine and 30 mL of saline) was injected. The needle was connected via a long IV tubing to a bag containing 500 ml of normal saline placed in a reusable pressure infusor, and normal saline was then injected to achieve progressive distension of the capsule, making the axillary and subscapular recesses more visible. Distension was continued until capsular rupture occurred; this was detected as a loss of resistance and contrast leakage and usually occurred after the injection of 10 to 55 ml of normal saline. The needle was then removed.

#### 2.5.2. Manipulation Therapy

**Figure 2:**
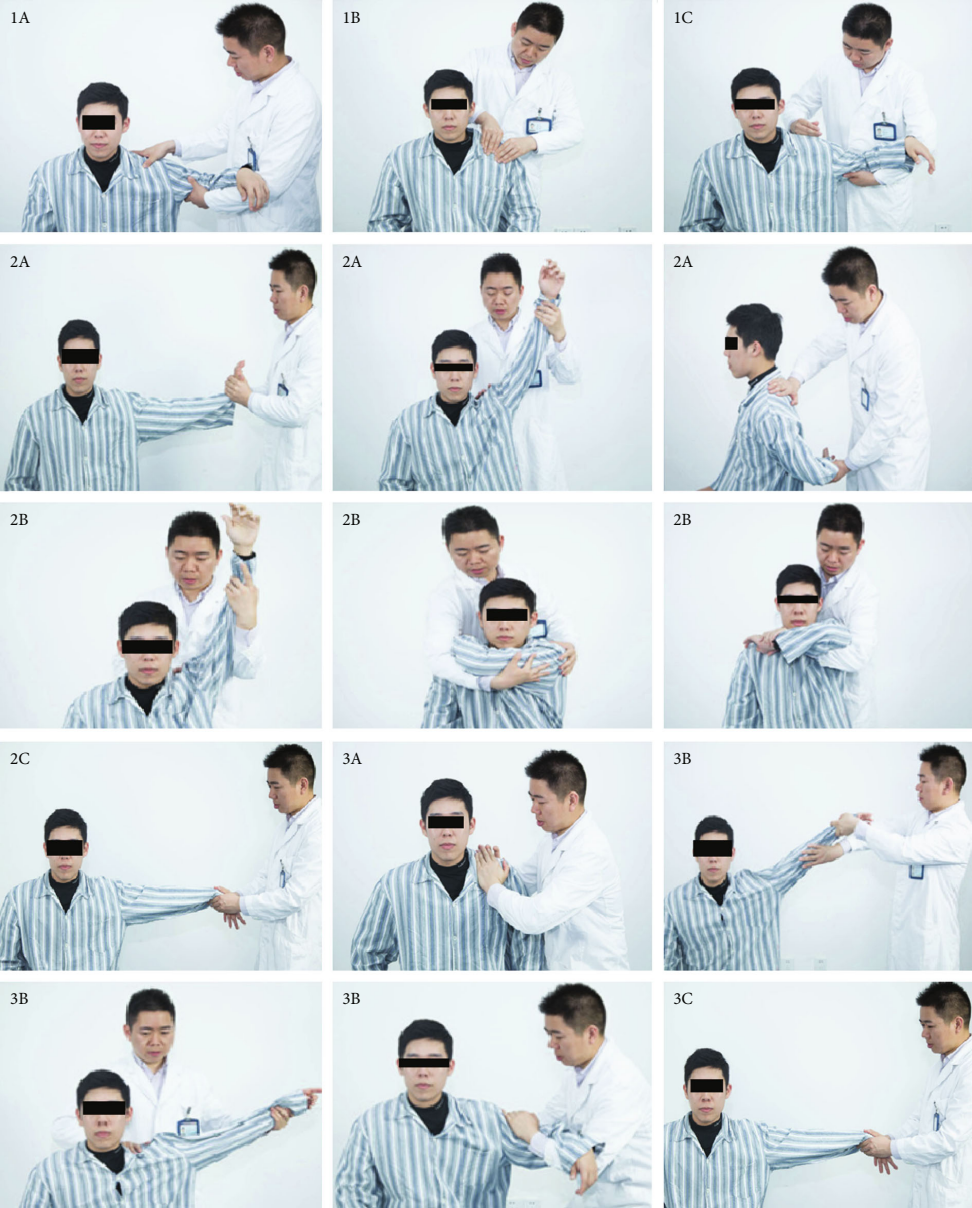
The detailed manipulation steps of Three-step and Nine-therapy manipulative therapy, which involves kneading, drawing, and rubbing the shoulder. (1a–1c) Soothing tendon step: the therapist kneaded the patient's lateral trapezius muscle, the superior muscle, and the deltoid muscle. (2a–2c) Osteopathic step: the therapist draws the patient's shoulder out of the exhibition position, the range from small to large, lifts the affected limb from the elbow and extends it then draws out the internal and external rotation of the affected shoulder to the back. (3a–3c) Dredging collateral step: the therapist rub shoulders with palms, buckle the wrist to rotate the shoulder on the outside booth then hold the wrist with both hands and shake shoulders.

All shoulder manipulations were performed by the senior author. The treatment group underwent manipulation after resting for 5 minutes following arthrographic distension.  Figure 2 shows the detailed manipulation steps.

1. Soothing tendon step

The therapist kneaded the patient's lateral trapezius muscle, supraspinatus muscle, and deltoid muscle.

(2) Osteopathic step

The therapist moved the patient's shoulder out of the exhibition position, lifted the affected limb from the elbow and extended it, and then performed internal and external rotation of the affected shoulder to the back.

(3) Dredging collateral step

The therapist used their palms to rub the patient's shoulders, buckled the wrist to rotate the shoulder to the outside, then held the patient's wrists with both hands, and shook the patient's shoulder.

#### 2.5.3. Additional Exercises

After the first injection, the patients were taught how to perform exercises to increase the joint ROM, including stretching forward and bending down to a desk, Codman exercises, and wall-climbing exercise with the fingers. Patients were instructed to regularly practice these exercises at home. At each hospital visit, the clinicians encouraged the patient to keep performing these exercises.

Both groups received a simple exercise program comprising pendulum exercises and a scapular setting (isometric scapular retraction). Participants were asked to stop taking any nonsteroidal anti-inflammatory drugs but were allowed paracetamol and codeine preparations. Patients were not permitted to receive other manual treatments (for example, physiotherapy, massage, and chiropractic treatment) or medical interventions (for example, intra-articular steroid injection) during the study.

### 2.6. Outcome Measures

Patient hospital visits occurred at baseline and at weeks 1, 2, 12, and 24 after treatment. Clinical outcomes were measured using the Visual Analog Scale (VAS) for shoulder pain, range of active motion, the SPADI, and Constant-Murley (CM) score. The VAS comprises a horizontal, 100 mm long line with “no pain” on the left side (score: 0) and “pain as bad as it could be” on the right side (score: 10). Patients were asked to place a hatch mark on the line that corresponded to their current level of pain. The VAS score was then determined by measuring the distance in millimeter between the left endpoint and the point that the patient had marked [18, 19]. The SPADI is a self-reported questionnaire developed to measure the pain and disability associated with shoulder pathology [14]; it includes five items regarding shoulder pain and eight items regarding the interference with daily life caused by shoulder disability. The CM score uses a total of 100 points to assess the shoulder, with a maximum of 10 points designated for internal rotation, external rotation, lateral elevation, forward elevation, and positioning of the arm, respectively, and a maximum of 10 points awarded for the ability to perform activities of daily living. The CM score also assesses pain (maximum of 15 points) and strength (maximum of 25 points) [20].

### 2.7. Statistical Analysis

The statistical analysis was performed by a statistician blinded to the group allocation. The SPSS 20.0 statistical software (SPSS Inc., Chicago, IL) was used for the statistical analysis. Continuous variables were presented as mean, standard deviation, median, quartiles, and interquartile ranges, while categorical variables were presented as frequency. The Pearson chi-square test was used to compare the qualitative variables. The Kolmogorov-Smirnov and Shapiro-Wilk tests were used to assess normally distributed variables. The Mann-Whitney *U* test was used to compare the groups, whereas the Friedman test was used for comparisons between timepoints. A *P* value of less than 0.05 was considered significant with a two-sided 90% confidence interval. An intention-to-treat analysis was performed, and missing data were imputed with the last observed response carried forward for all measures using the “last-value-carried-forward” principle [21].

### 2.8. Quality Control

To avoid selection bias, a blinded investigator verified the eligibility criteria and study enrollment. During the trial, supervisors checked case report forms and interventions. Dropouts, withdrawals (and reasons), and compliance of all patients were recorded in detail throughout the treatment and follow-up periods.

### 2.9. Safety Assessments

Participants remained in the hospital for 30 minutes after each injection to be monitored for any signs of acute adverse reactions. At each follow-up visit, participants were checked for late adverse reactions, such as subcutaneous fat atrophy, skin depigmentation, and infection.

## 3. Results

### 3.1. Patient Demographic and Clinical Characteristics

Database records of patients treated at one of the six participating centers for symptomatic FS between Apr 2013 and Mar 2015 were prospectively collected and analyzed. Of the 228 patients enrolled in this study, 48 were excluded because they did not meet the eligibility criteria. The remaining 180 patients who agreed to participate were randomized into the treatment group (distension arthrography combined with manipulation) or the control group (only distension arthrography). The cohort comprised 56 men and 124 women aged 42–65 years. The mean patient ages in the treatment and control groups were 54.97 and 52.21 years, respectively. The duration of symptoms ranged from 1 to 24 months, and the mean durations of symptoms in the treatment and control groups were 5.26 and 6.46 months, respectively. The baseline ROM of the shoulder, SPADI, and CM score at presentation were collected and analyzed.  Table 1 summarizes the demographic and clinical characteristics. The characteristics did not significantly differ between groups.

### 3.2. Improvements in Clinical Symptoms

Table 2 shows the outcome measures in both groups. After the first treatment, the VAS score, ROM, SPADI, and CM score were significantly improved in both groups. Compared with the control group, the treatment group had a significantly better curative effect regarding the VAS score, SPADI, abduction function, extorsion function, and uplift function. However, there was no difference between groups in the improvement of the adduction function, intorsion function, posterior extension function, and CM score.

After the second treatment, the VAS score, ROM, SPADI, and CM score were significantly improved in both groups. Compared with the control group, the treatment group had a significantly better curative effect regarding the VAS score, SPADI, CM score, abduction function, adduction function, and uplift function. However, there was no significant difference between groups in the improvement of the intorsion function, extorsion function, and posterior extension function.

At the 12- and 24-week follow-up visits, the VAS scores and SPADI were significantly improved in both groups. The treatment group achieved significantly better improvements than the control group (Table 3). DAM is effective for the long-term relief of pain and disability in patients with frozen shoulder.

### 3.3. Safety Monitoring of DAM

No obvious adverse events occurred in all patients who participated in the clinical trial. Some patients complained of more shoulder pain and discomfort during the treatment, but they were still within the patient's tolerance range and finally successfully completed the treatment.

## 4. Discussion

The results of the present study confirmed the superiority of distension arthrography plus manipulation therapy over distension arthrography alone. Distension arthrography comprises the stable and uniform injection of dilation fluid into the shoulder joint cavity to distend the articular capsule without causing excessive damage. Injecting a large amount of liquid into the articular capsule dilutes the accumulated acid metabolites and pain-causing substances in the joint to achieve analgesia. After distension arthrography, shoulder manipulation releases the adhesions and remits the spasm of muscle.

A previous study confirmed the effectiveness of distension arthrography in treating patients with painful restriction of more than 30° during shoulder passive range of motion for longer than 3 months [22]. The shoulder ROM of the patients included in the present study was similar to that of the patients included in the previous study, but the course of the disease was less than 6 months. Distension arthrography alone may not completely release the adhesions in patients with a protracted course of disease or in those with severe joint capsule adhesions.

There are several issues regarding distension arthrography that need to be resolved. First, the appropriate volume of dilation fluid is unclarified. Ogul et al. [23] found that the mean total glenohumeral joint volume was 22.52 ± 1.1 cm^3^ in the patient group and 26.01 ± 1.2 cm^3^ in the control group. If the joint dilator volume is too large, the joint capsule is likely to rupture. Kim et al. [24] reported that distension arthrography achieves a better effect when the joint capsule remains intact. Their study found that with repeated distension arthrography, the maximum volume increased (from 18.8 ± 7.3 ml to 24.2 ± 7.0 ml) while the maximum pressure decreased, indicating joint capsule rupture [24]. We set the expansion dose at 35 ml based on the existing literature and clinical experience. Further research is needed to determine the appropriate dilation fluid volume to maximize the curative effect.

The second issue is the optimal number of distension arthrography treatments. Fouquet et al. [25, 26] reported that the first treatment achieves the most marked improvement in symptoms, while the second treatment achieves a significantly reduced degree of improvement, and the third treatment achieves a very small improvement in joint function. Related studies in which patients were treated with distension arthrography once [25, 26] and three times [27] showed that the first treatment achieves the most marked improvement in symptoms, but that only one treatment is insufficient. A second treatment is necessary to further improve the symptoms. However, too many distension arthrography treatments achieve little effect and increase the risk of infection. We conclude that a third treatment should only be considered for the few patients with unsatisfactory functional recovery after two treatments.

The third issue with distension arthrography concerns the selection of the optimal injection solution. There is no uniform formula for the expansion fluid in distension arthrography. We use normal saline and lidocaine. Injecting a large amount of normal saline into the joint cavity of the shoulder dilates the joint cavity and dilutes the accumulated acid metabolites and pain-causing substances. Lidocaine results in local anesthesia, relieving pain and making the dilation process and manipulation easier.

Although there is reportedly no significant difference in the efficacy of distension arthrography versus intra-articular injection in the treatment of shoulder periarthritis [28], distension arthrography achieves a reliable therapeutic effect and significantly improves the function and ROM of the shoulder in the long-term [29]. Further study is needed to determine whether distension arthrography achieves clinically better improvements than intra-articular injection alone.

The present study showed that distension arthrography effectively reduced shoulder pain and improved the movement and function of the shoulder joint. Furthermore, distension arthrography combined with manipulation therapy more effectively improved the shoulder pain, function, and movement of patients with FS than distension arthrography alone. The treatment of FS with distension arthrography combined with manipulation therapy is safe and is worthy of popularization and application.

There is a lack of evidence regarding the appropriate distension arthrography pressure. Furthermore, the body surface positioning used for injection may cause inaccurate injection. In future studies, we hypothesize that accurate injection using ultrasound guidance would result in improvements in passive shoulder ROM, general clinical outcome measures, and pain relief.

## 5. Limitations

This pilot study has a number of limitations. First, due to the long time span of this research, some subcenters have replaced researchers. And some subcenters are not strong enough to execute the research plan. Second, the patients may have received other treatments before and after enrollment, which may affect the results of the experiment. Another limit of the study is that the injection is not guided to be more accurate. Finally, statistically significant results may not be clinically relevant.

## 6. Conclusion

Distension arthrography significantly improved the symptoms of FS in the short- to medium-term. The clinical efficacy was further improved by the addition of manual therapy. In summary, the combination of distention arthrography and manual therapy achieved better therapeutic effects than distention arthrography alone.

## Figures and Tables

**Table 1:** Demographic and clinical characteristics of the patients.

Variable	Treatment group (*n* = 90)	Controlled group (*n* = 90)	*P* value
Age (year) ± SD	54.97 ± 4.8	52.21 ± 7.77	0.993
Gender: male *n* (%)	23 (25.6)	33 (36.7)	0.107
Duration of symptoms (months) ± SD	5.26 ± 0.96	6.46 ± 0.57	0.600
Affected side (left) *n* (%)	47 (52.2)	57 (63.3)	0.131
VAS	7.43 ± 1.25	7.31 ± 1.30	0.504
Abduction	86.86 ± 21.05°	91.73 ± 21.70°	0.081
Flexion	29.22 ± 12.00°	28.38 ± 14.90°	0.104
External rotation	38.21 ± 17.31°	35.29 ± 18.31°	0.508
Internal rotation	39.73 ± 19.38°	37.71 ± 20.80°	0.173
Elevation	97.01 ± 20.90°	99.78 ± 22.48°	0.542
Posterior extension	25.66 ± 10.06°	25.49 ± 10.17°	0.980
SPADI score	66.90 ± 13.96	66.10 ± 14.51	0.725
Constant-Murley score	54.14 ± 11.16	53.51 ± 12.62	0.682

The results are expressed as mean ± SD (standard deviation) for quantitative variables and as frequency (numbers and percent) for categorized variables. SPADI: Shoulder Pain and Disability Index; VAS: Visual Analog Scale.

**Table 2:** Comparison of ROM and CM score between groups and within groups.

Item	Group	Baseline	1 week	2 weeks
Abduction	Treatment	86.86 ± 21.05°	117.23 ± 34.89°^∗^	133.43 ± 38.47°^∗^
	Controlled	91.73 ± 21.70°	114.76 ± 32.04°^∗^	124.90 ± 32.58°^∗^
	*P*	0.081	0.045	0.003
Flexion	Treatment	29.22 ± 12.00°	38.94 ± 13.40°^∗^	45.69 ± 16.25°^∗^
	Controlled	28.38 ± 14.90°	37.21 ± 13.92°^∗^	41.52 ± 13.08°^∗^
	*P*	0.104	0.299	0.023
External rotation	Treatment	38.21 ± 17.31°	51.43 ± 19.67°^∗^	64.08 ± 22.09°^∗^
	Controlled	35.29 ± 18.31°	45.77 ± 19.31°^∗^	55.55 ± 18.33°^∗^
	*P*	0.058	0.049	0.070
Internal rotation	Treatment	39.73 ± 19.38°	51.65 ± 15.34°^∗^	62.89 ± 12.85°^∗^
	Controlled	37.71 ± 20.80°	51.44 ± 15.99°^∗^	60.27 ± 11.95°^∗^
	*P*	0.173	0.921	0.237
Upthrow	Treatment	97.01 ± 20.90°	131.64 ± 28.79°^∗^	148.65 ± 28.74°^∗^
	Controlled	99.78 ± 22.48°	123.65 ± 30.34°^∗^	135.94 ± 30.01°^∗^
	*P*	0.542	0.004	<0.001
Rear protraction	Treatment	25.66 ± 10.06°	37.31 ± 10.83°^∗^	44.96 ± 13.74°^∗^
	Controlled	25.49 ± 10.17°	37.23 ± 10.88°^∗^	42.88 ± 13.18°^∗^
	*P*	0.980	0.616	0.610
Constant-Murley score	Treatment	54.14 ± 11.16	69.41 ± 12.82^∗^	79.22 ± 13.45^∗^
	Controlled	53.51 ± 12.62	65.84 ± 15.94^∗^	71.73 ± 14.50^∗^
	*P*	0.682	0.061	0.002

^∗^There was a statistically significant difference before and after treatment, *P* value < 0.05; CM: Constant-Murley score.

**Table 3:** Comparison of VAS and SPADI score between groups and within groups.

Item	Group	Baseline	1 week	2 weeks	12 weeks	24 weeks
VAS	Treatment	7.43 ± 1.25	4.28 ± 1.94^∗^	3.03 ± 2.02^∗^	1.52 ± 1.20^∗^	0.88 ± 0.90^∗^
	Control	7.31 ± 1.30	4.89 ± 2.05^∗^	3.78 ± 1.84^∗^	2.27 ± 1.46^∗^	1.74 ± 1.35^∗^
	*P*	0.504	0.006	0.004	<0.001	0.001
SPADI	Treatment	66.90 ± 13.96	39.13 ± 16.10^∗^	25.45 ± 16.63^∗^	15.09 ± 9.30^∗^	10.49 ± 6.81^∗^
	Control	66.10 ± 14.51	44.68 ± 18.94^∗^	35.32 ± 16.31^∗^	26.92 ± 13.53^∗^	20.66 ± 11.18^∗^
	*P*	0.725	0.002	0.002	<0.001	<0.001

^∗^There was a statistically significant difference before and after treatment, *P* value < 0.05; SPADI: Shoulder Pain and Disability Index; VAS: Visual Analog Scale.

## Data Availability

Data are available on request.
